# A free amino acid‐based diet partially prevents symptoms of cow's milk allergy in mice after oral sensitization with whey

**DOI:** 10.1002/iid3.288

**Published:** 2020-02-07

**Authors:** Joris H. J. van Sadelhoff, Astrid Hogenkamp, Selma P. Wiertsema, Lucien F. Harthoorn, Reinilde Loonstra, Anita Hartog, Johan Garssen

**Affiliations:** ^1^ Division of Pharmacology, Utrecht Institute for Pharmaceutical Sciences, Faculty of Science Utrecht University Utrecht The Netherlands; ^2^ CeO Immunology Danone Nutricia Research Utrecht The Netherlands

**Keywords:** allergy, free amino acids, infant milk formula, prevention

## Abstract

**Background:**

Amino acid‐based formulas (AAFs) are used for the dietary management of cow's milk allergy (CMA). Whether AAFs have the potential to prevent the development and/or symptoms of CMA is not known.

**Objective:**

The present study evaluated the preventive effects of an amino acid (AA)‐based diet on allergic sensitization and symptoms of CMA in mice and aimed to provide insight into the underlying mechanism.

**Methods:**

C3H/HeOuJ mice were sensitized with whey protein or with phosphate‐buffered saline as sham‐sensitized control. Starting 2 weeks before sensitization, mice were fed with either a protein‐based diet or an AA‐based diet with an AA composition based on that of the AAF Neocate, a commercially available AAF prescribed for the dietary management of CMA. Upon challenge, allergic symptoms, mast cell degranulation, whey‐specific immunoglobulin levels, and FoxP3^+^ cell counts in jejunum sections were assessed.

**Results:**

Compared to mice fed with the protein‐based diet, AA‐fed mice had significantly lower acute allergic skin responses. Moreover, the AA‐based diet prevented the whey‐induced symptoms of anaphylaxis and drop in body temperature. Whereas the AA‐based diet had no effect on the levels of serum IgE and mucosal mast cell protease‐1 (mMCP‐1), AA‐fed mice had significantly lower serum IgG2a levels and tended to have lower IgG1 levels (*P* = .076). In addition, the AA‐based diet prevented the whey‐induced decrease in FoxP3^+^ cells. In sham‐sensitized mice, no differences between the two diets were observed in any of the tested parameters.

**Conclusion:**

This study demonstrates that an AA‐based diet can at least partially prevent allergic symptoms of CMA in mice. Differences in FoxP3^+^ cell counts and serum levels of IgG2a and IgG1 may suggest enhanced anti‐inflammatory and tolerizing capacities in AA‐fed mice. This, combined with the absence of effects in sham‐sensitized mice indicates that AAFs for the prevention of food allergies may be an interesting concept that warrants further research.

## Graphical abstract

This study demonstrates that an amino acid‐based diet can at least partially prevent clinical symptoms of cow's milk allergy (CMA) in mice. Differences in FoxP3^+^ cell counts and serum levels of IgG2a and IgG1 suggest enhanced anti‐inflammatory and tolerizing capacities in mice fed the amino acid‐based diet.

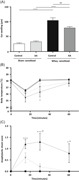

## 1 INTRODUCTION

Cow's milk allergy (CMA) is one of the most commonly occurring food allergies in infancy, affecting up to 3% of the children at 1 year of age in developed countries.^1^, ^2^ CMA in infancy represents an increasing global health and economic burden, which is caused not only by an increased prevalence over the last decades, but also by an increased persistence, severity, and complexity of the condition.^3^, ^4^, ^5^, ^6^ Following ingestion of cow's milk, affected children usually present moderate symptoms involving the skin, the gastrointestinal, and/or the respiratory tract, but life‐threatening systemic anaphylaxis may also occur.^7^, ^8^ In fact, cow's milk is described as one of the most common foods capable of inducing fatal anaphylactic reactions in infancy.^9^, ^10^ In addition to these acute clinical manifestations, CMA in early life can also have long‐lasting effects, including delays in growth and development,^11^, ^12^ as well as increased risk of developing atopic diseases later in life.^13^, ^14^ Hence, strategies to suppress or prevent the development of CMA are of major importance.

To date, the standard dietary management of CMA in children is allergen avoidance through elimination of cow's milk from the diet.^15^ Without appropriate substitution, however, such an elimination diet may lead to nutritional deficiencies and poor growth.^16^ A variety of formulas have been developed and acknowledged to be suitable substitutions for cow's milk. Extensively hydrolyzed formulas (eHFs) are recommended for infants with mild CMA, whereas for infants with severe CMA and for infants who either do not tolerate eHFs or for whom eHFs fail to resolve CMA symptoms, amino acid‐based formulas (AAFs) are recommended.^17^ Besides being nutritionally adequate,^18^ AAFs are consistently demonstrated to provide relief and a faster recovery from symptoms of CMA, including gastrointestinal (eg, vomiting and diarrhea) and skin conditions (eg, atopic dermatitis).^11^, ^19^, ^20^, ^21^ Moreover, nutritional intervention with an AAF has been shown to fully normalize the growth of patients with CMA.^22^, ^23^ Studies also show that intake of AAFs is very well‐tolerated, safe, and has no long‐term effects on protein‐metabolism.^22^, ^23^, ^24^, ^25^ In summary, AAFs are proven to be an effective and safe way of dietary management of CMA.

Whereas numerous studies have investigated AAFs as a dietary management option for CMA, little is known about the potential of AAFs to prevent allergic sensitization and clinical symptoms of CMA. AAFs have been demonstrated to exert anti‐inflammatory effects both in human and in in vitro immune models, which may inhibit allergic sensitization.^26^, ^27^ In infants with non‐IgE‐mediated CMA, an AAF prescribed for dietary management of CMA (ie, Neocate) reduced levels of proinflammatory cytokines interleukin‐6 (IL‐6) and tumor necrosis factor‐α (TNF‐α), which are both indicated to drive allergic sensitization.^26^, ^28^, ^29^ This reduction was accompanied by a significant decline in epithelial‐derived interleukin‐33 (IL‐33) and in T‐cell helper type 2 (T_H_2)‐associated cytokines IL‐4 and IL‐13, which are also known to promote allergic sensitization.^30^ Based on these findings, it was hypothesized that AAFs may prevent the development of CMA. Therefore, the present study evaluated the preventive effects of an AA‐based diet on allergic sensitization and allergic symptoms of CMA, using an extensively validated murine model of orally induced CMA.^31^

## 2 METHODS

### 2.1 Animals, diets, and consumption measurement

Four‐week old female, specific pathogen‐free C3H/HeOuJ mice, which were bred and raised for at least two generations on a cow's milk protein‐free diet were purchased from Charles River Laboratories (Maastricht, The Netherlands). All mice were housed in filter‐topped makrolon cages (n = 5 per cage) on a 12‐hour light/dark cycle with unlimited access to food and water at the animal facility of Utrecht University and were acclimatized for 7 days. Animal care and use was performed in strict accordance with the principles of good laboratory animal care as stated by the European Directive 2010/63/EU for the protection of animals used for scientific purposes. All experimental procedures were approved by an independent ethics committee for animal experimentation (DEC Consult, Soest, The Netherlands).

**Figure 1:**
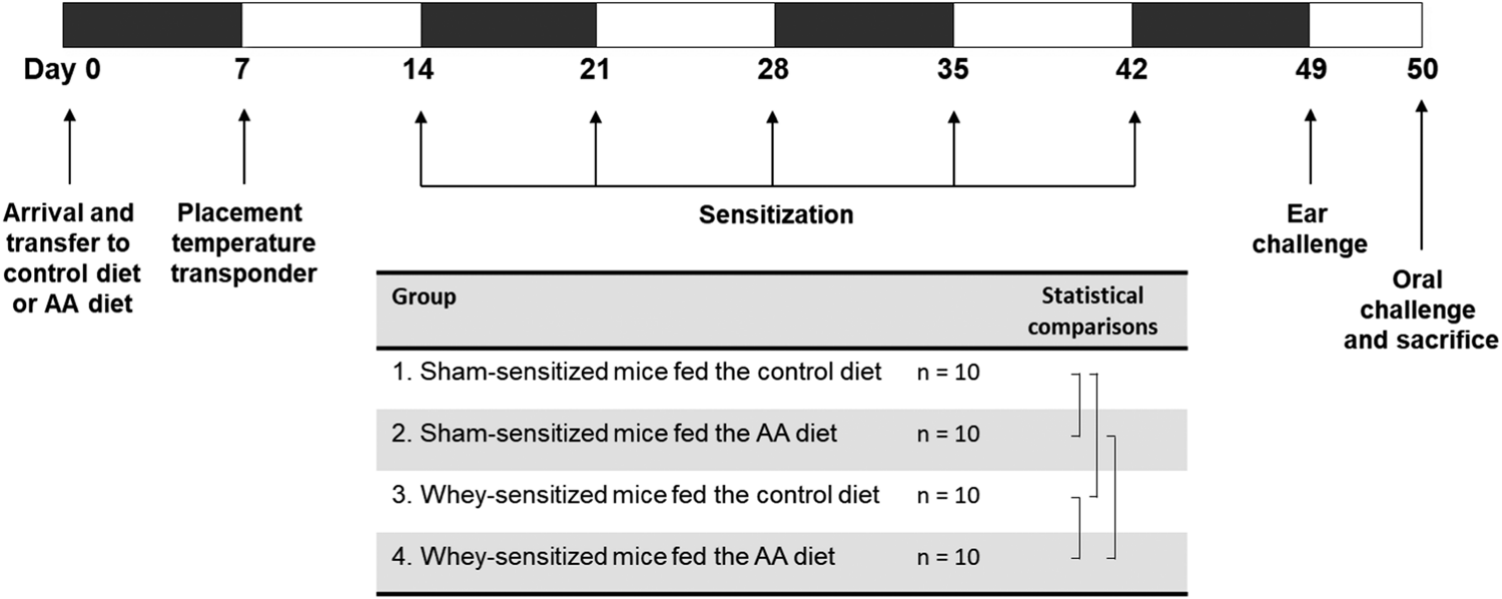
Schematic representation of the experimental model and the experimental groups

Mice were fed ad libitum either a control, semi‐purified AIN‐93G soy protein‐based diet (SSniff Spezialdiaten GmbH, Soest, Germany),^32^ or an experimental AA‐based diet with an AA composition based on that of the commercially available AAF Neocate (Nutricia; Table 1). Other than the protein content, the diets were identical in all nutrients. Two weeks before the first sensitization, mice were placed on either of the two diets until the end of the study protocol (Figure 1). For consumption measurement, food was weighed per cage with intervals of 3 to 4 days when food was refreshed. The body weight of all mice was measured at study days 1, 2, 3, 4, 5, 6, 8, 13, 20, 27, 34, 41, and 48. Throughout the entire duration of the study, the welfare of the animals was carefully monitored through observation of appearance, body functions (eg, changes in body temperature) and behaviors.

**Table 1:** Composition of the protein‐based control diet and the amino acid‐based experimental diet

Components	Control diet, g/kg	Amino acid diet, g/kg
**Carbohydrates**		
Corn starch	397.5	397.5
Dextrinized corn starch	132.0	132.0
Sucrose	100.0	100.0
**Fiber**		
Cellulose	50.0	50.0
**Protein/Amino acids**		
Soy protein	200.0	–
Free amino acids	3.0	203.0
L‐Alanine	–	8.0
L‐Arginine	–	14.2
L‐Aspartic acid	–	13.2
L‐Cysteine	1.0	5.2
L‐Glutamine	–	21.5
L‐Glycine	–	12.5
L‐Histidine	–	8.1
L‐Isoleucine	–	12.5
L‐Leucine	–	21.3
L‐Lysine	–	14.6
L‐Methionine	–	3.4
L‐phenylalanine	–	9.6
L‐Proline	–	11.2
L‐Serine	–	9.3
L‐Threonine	–	10.5
L‐Tryptophan	–	4.2
L‐Tyrosine	–	9.6
L‐Valine	–	13.6
L‐Carnitine	–	0.1
Taurine	–	0.4
DL‐methionine	2.0	–
**Fat**		
Soybean oil	70.0	70.0
**Other**		
Mineral mix	35.0	35.0
Vitamin mix	10.0	10.0
Choline bitartrate	2.5	2.5
Tert‐butylhydroquinone	0.014	0.014

### 2.2 Experimental animal procedures

Upon arrival, 40 mice were randomly assigned to one of the following four groups (n = 10 per group): (a) a sham‐sensitized control group fed with the soy protein‐based diet, (b) a sham‐sensitized experimental group fed with the AA diet, (c) a whey‐sensitized control group fed with the soy protein‐based diet, and (d) a whey‐sensitized experimental group fed with the AA diet. Whey‐sensitized mice were sensitized orally via gavage with 20 mg of homogenized whey (WPC60; Milei, Friesland Campina) in 500 µL phosphate‐buffered saline (PBS), containing 10 µg cholera toxin (CT) (List Biological Laboratories) as an adjuvant.^33^ Sham‐sensitized mice received 10 µg CT in 500 µL PBS. Mice were sensitized once a week for 5 consecutive weeks, starting at day 14 (Figure 1).

One week after the final sensitization, all mice received an intradermal (i.d.) injection of 10 µg homogenized whey in 10 µL PBS in the pinnae of both ears to measure the acute allergic skin reaction as the primary study outcome. Ear thickness was measured in duplicate for each ear before and 1 hour after i.d. injection with whey, using a digital micrometer (Mitutoyo). Whey‐induced ear swelling was expressed as delta (Δ) µm by subtracting the basal ear thickness from the ear thickness measured at 1 hour after i.d. injection. Moreover, at 15, 30, 45, and 60 minutes after the i.d. injection, the body temperature of the mice was measured using temperature transponders (IPTT‐300; Biomedical data systems), which were injected subcutaneously 7 days before the sensitization started (Figure 1). In addition to the measurement of body temperature, anaphylactic shock severity was scored using a validated 0‐ to 5‐point scoring system (Table 2) adapted from Li et al.^34^ Animals reaching a shock score of 4 (n = 1), considered as the humane endpoint, were euthanized and not considered for further analysis.

**Table 2:** Scoring system for symptoms of systemic anaphylaxis

Score	Symptoms
0	No symptoms
1	Scratching and rubbing around the nose and head
2	Puffiness around the eyes and mouth, pilar erecti, reduced activity, and/or decreased activity with increased respiratory rate
3	Wheezing, labored respiration, and cyanosis around the mouth and the tail
4	No activity after prodding or tremor and convulsion
5	Death

One day after the measurement of the acute allergic skin reaction, the mice were challenged intragastrically (i.g.) with 100 mg homogenized whey in 500 µL PBS. After 30 minutes, blood samples were taken via orbital extraction under terminal anesthesia (isoflurane/air), followed by cervical dislocation. Sera were stored at −80°C for immunoglobulins (Igs) and mouse mast cell protease‐1 (mMCP‐1) analyses.

### 2.3 Measurement of serum levels of allergen‐specific immunoglobulins and mMCP‐1

Levels of whey‐specific IgE, IgG1, and IgG2a were determined by enzyme‐linked immunosorbent assay (ELISA) in serum collected after orbital extraction. Microlon plates (Greiner) were coated with 100 μL whey (20 μg/mL) in carbonate/bicarbonate coating buffer (0.05M, pH = 9.6; Sigma‐Aldrich) for 18 hours at 4°C. Subsequently, plates were washed and blocked for 1 hour with 2% human serum albumin in PBS. Serum samples were incubated for 2 hours at room temperature (RT), after which plates were washed and incubated with 1 μg/mL biotin‐labeled rat anti‐mouse IgE, IgG1, or IgG2a (BD Pharmingen) in PBS for 90 minutes at RT. Plates were subsequently washed, incubated with 0.5 μg/mL streptavidin‐horseradish peroxidase (Sanquin) in PBS for 1 hour at RT, and developed with tetramethyl benzidine substrate (Pierce; Thermo Fisher Scientific). After 10 minutes, the reaction was stopped with 4M H_2_SO_4_ and absorbance was measured at 450 nm with a microplate reader (Powerwave HT; BioTek). The results are expressed as arbitrary units (AUs) with pooled sera from whey‐alum‐immunized mice serving as positive reference to compose a titration curve.

Concentrations of mMCP‐1 in serum were determined using a commercially available ELISA kit (eBioscience), according to the manufacturer's instructions.

### 2.4 Immunohistochemistry for Forkhead box P3

The small intestine of each mouse was dissected, and the jejunum was fixed in 4% formaldehyde in PBS for 24 hours at RT, dehydrated and subsequently embedded in paraffin. Sections of 5 μm were cut using a microtome (Leica Microsystems) and stained for intracellular Forkhead box P3 (FoxP3) expression. Paraffin sections were dewaxed and boiled for 12 minutes in 0.01M sodium citrate buffer (pH = 6.0). Next, sections were washed in demineralized water and endogenous peroxidase activity was blocked by incubation with 3% H_2_O_2_ in methanol for 15 minutes at RT. Sections were washed, blocked for 90 minutes with 5% normal rabbit serum (Dako) in PBS with 3% bovine serum albumin (BSA), washed again and incubated overnight at 4°C with rat anti‐mouse FoxP3 purified antibody (12.5 μg/mL, 14‐5773; eBioscience) or rat IgG2a isotype (2.5 μg/mL, 14‐4321; eBioscience) in 3% BSA/PBS as a control. After washing, slides were incubated with a biotinylated rabbit‐anti‐rat antibody (5 μg/mL, 312‐065‐003; Jackson ImmunoResearch) in 3% BSA/PBS and 1% normal mouse serum (Invitrogen) and subsequently incubated with avidin biotin complex (Vectastain Elite ABC Kit; Vector Laboratories) in 3% BSA/PBS for 1 hour at RT. The staining was visualized using 0.05% 3,3′‐diaminobenzidine tetra‐hydrochloride (DAB; Sigma‐Aldrich) in Tris buffer (pH = 7.6) for 8 minutes followed by counterstaining of the sections with haematoxylin (Merck Millipore, Amsterdam, The Netherlands). Next, sections were dehydrated and covered with Pertex mounting medium (Histolab) and a cover glass. FoxP3‐positive (FoxP3^+^) cells were counted only in completely attached villi and were counted blindly and in duplicate by two independent scientists. Results are expressed as FoxP3^+^ cells per villi. Graphic images were taken with an Olympus BX50F microscope equipped with a Leica DFC320 digital camera.

### 2.5 Statistical analysis

Experimental results are expressed as means ± standard error of the mean (SEM). Normal distribution was tested for each readout using the D'Agostino & Pearson normality test. Differences between preselected pairs (n = 4; Figure 1) were analyzed by means of a one‐way analysis of variance (ANOVA) with a post‐hoc Bonferroni test to correct for multiple comparisons. Levels of mMCP‐1 and whey‐specific Igs in serum were log‐transformed to obtain normality. Anaphylactic shock scores did not obtain normality and hence were analyzed using a Kruskal–Wallis test with a post‐hoc Dunn's test. Correlations of Ig serum levels and clinical parameters were calculated by Pearson Correlation when data were normally distributed or by Spearman Rho Correlation when data were not normally distributed. All repeated measurements of the anaphylactic shock score were taken into account simultaneously when testing for correlations, by using the area under the curve (AUC) of this parameter as calculated using the measurements at 0, 15, 30, and 60 minutes after i.d. challenge. All calculations and statistical analyses were performed using GraphPad Prism Software (version 7). *P* < .05 were considered statistically significant. Trends were indicated when *P* < .10.

## 3 RESULTS

### 3.1 The amino acid‐based diet reduces the allergic skin response and prevents anaphylactic symptoms in response to whey protein

**Figure 2:**
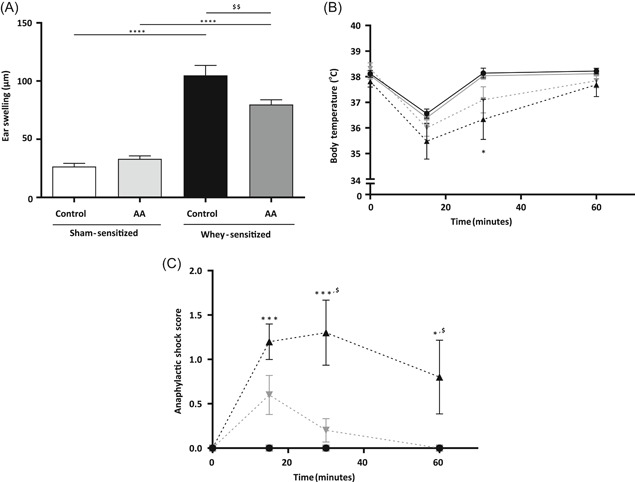
The acute allergic skin response, body temperature, and anaphylactic shock severity of mice fed with the control or with the amino acid (AA)‐based diet. A, Ear swelling was measured before and 1 hour after i.d. challenge with whey. Body temperature (B) and anaphylactic shock severity (C) was measured before and 15, 30, and 60 minutes after i.d. challenge. Groups are as follows: 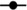 sham‐sensitized mice fed with the control diet (n = 10); 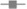 sham‐sensitized mice fed with the AA diet (n = 10); 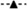 whey‐sensitized mice fed with the control diet (n = 9); 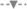 whey‐sensitized mice fed with the AA diet (n = 10). Values are expressed as mean ±  standard error of the mean (SEM). Significant differences between whey‐sensitized and sham‐sensitized mice are indicated by *P < .05, **P < .01, ***P < .001, and ****P < .0001. Differences between whey‐sensitized mice fed with the control diet and those fed with the AA diet are indicated by ^$^ *P* < .05, ^$$^ *P* < .01. Differences are analyzed with a one‐way analysis of variance (ANOVA) followed by a Bonferroni post‐hoc test (A and B) or a Kruskal–Wallis test with a post‐hoc Dunn's test (C)

One hour after the i.d. challenge, the ear swelling of whey‐sensitized mice fed with either the control or the AA diet was significantly higher than that of diet‐matched sham‐sensitized mice (Figure 2A). A significantly lower ear swelling was found in whey‐sensitized mice fed with the AA diet compared to whey‐sensitized mice fed with the control diet. In sham‐sensitized mice, no differences in ear swelling were observed between the two groups.

Body temperature and anaphylactic symptoms were assessed at 15, 30, and 60 minutes after the i.d. challenge. At 30 minutes after i.d. challenge, whey‐sensitized mice fed with the control diet had a significantly lower body temperature, an indication of (anaphylactic) shock, than sham‐sensitized mice fed with the control diet (Figure 2B). In contrast, no significant difference in body temperature was found at any of the indicated time points between whey‐sensitized AA‐fed mice and diet‐matched sham‐sensitized mice. Anaphylactic shock symptoms were scored as described above (Table 2). One of the whey‐sensitized mice fed with the control diet reached the humane endpoint and, hence, was euthanized and excluded from further analyses. At all time points, the anaphylactic shock score of whey‐sensitized mice fed with the control diet was significantly higher than that of sham‐sensitized mice fed with the control diet (Figure 2C). The AA diet prevented whey‐induced symptoms of anaphylaxis, as no significant difference in anaphylactic shock score was observed when comparing whey‐sensitized mice fed with the AA diet with diet‐matched sham‐sensitized mice at any of the time points. Compared to whey‐sensitized mice fed with the control diet, whey‐sensitized AA‐fed mice had significantly lower anaphylaxis scores at 30 and 60 minutes after i.d. challenge. No differences were observed in either body temperature or anaphylactic shock score between sham‐sensitized mice fed with the control diet and those fed with the AA diet.

### 3.2 The AA diet lowers serum levels of whey‐specific IgG1 and IgG2a, whereas whey‐specific IgE and mMCP‐1 levels are unaffected

**Figure 3:**
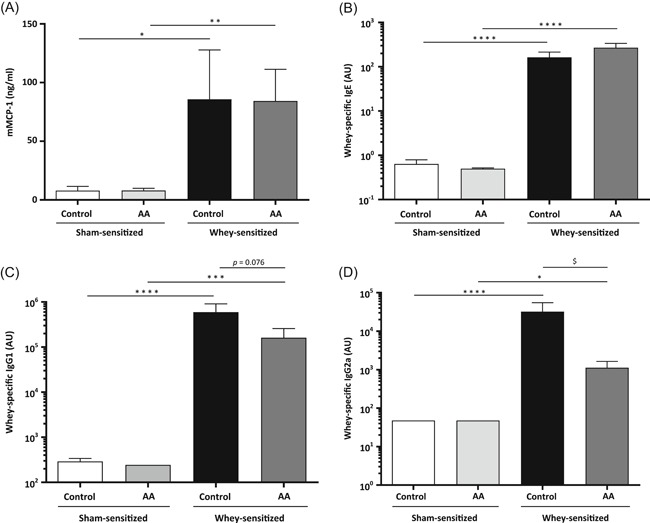
Serum levels of mouse mast cell protease‐1 (mMCP‐1) and whey‐specific Ig of mice fed a control diet or an AA‐based diet. Serum of all mice (n = 9‐10 per group) was harvested 30 minutes after the oral challenge. Serum levels are shown for (A) mMCP‐1 (ng/mL), (B) whey‐specific IgE (arbitrary unit [AU]), (C) whey‐specific IgG1 (AU), and (D) whey‐specific IgG2a (AU). Values are expressed as mean ± SEM. Significant differences between whey‐sensitized and sham‐sensitized mice are indicated by *P < .05, **P < .01, ***P < .001, and ****P < .0001. Differences between whey‐sensitized mice fed with the control diet and those fed with the AA diet are indicated by ^**$**^ *P* < .05. Differences were analyzed with a one^**‐**^way ANOVA followed by a Bonferroni post‐hoc test for selected groups after log‐transformation of the data. AA, amino acid; ANOVA, analysis of variance; SEM, standard error of the mean

Levels of mMCP‐1 and whey‐specific Igs were analyzed in mouse sera that were obtained 30 minutes after the oral challenge. The concentrations of mMCP‐1 and whey‐specific IgE, IgG1, and IgG2a were significantly higher in the sera of whey‐sensitized mice as compared to sham‐sensitized mice, both for mice fed with the control diet and for mice fed with the AA diet (Figure 3A‐D). No significant differences were observed in mMCP‐1 and whey‐specific IgE levels between mice fed with the control diet and those fed with the AA diet (Figure 3A,B). However, whey‐sensitized mice fed with the AA diet had a tendency towards lower levels of IgG1 (*P* = .076; Figure 3C) and had significantly lower levels of IgG2a (Figure 3D) compared to whey‐sensitized mice fed with the control diet. The mean serum levels of IgG1 and IgG2a in whey‐sensitized mice fed with the AA diet were 3.7 and 28.8 times lower, respectively, than those levels observed in whey‐sensitized mice fed with the control diet. No differences were observed in serum levels of any of the tested parameters between sham‐sensitized mice fed with the AA diet and those fed with the control diet.

### 3.3 Whey‐specific immunoglobulins positively correlate with the induction of allergic symptoms but only whey‐specific IgG1 and IgG2a correlate with the severity of allergic symptoms

**Figure 4:**
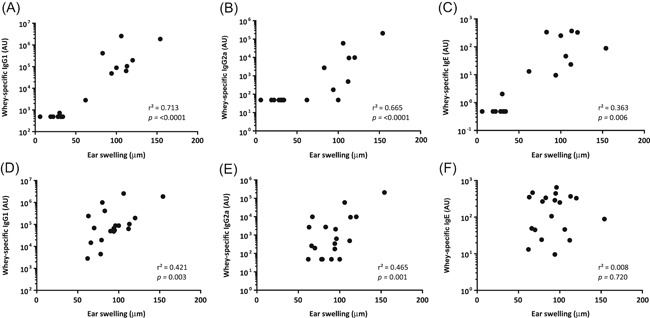
Correlations between Ig serum levels and the acute allergic skin response. Correlations are shown for ear swelling (µm) and serum levels of whey‐specific IgG1 (A), IgG2a (B), and IgE (C) in mice fed the control diet (n = 19). Moreover, correlations are shown for ear swelling (µm) and serum levels of whey‐specific IgG1 (D), IgG2a (E), and IgE (F) in all whey‐sensitized mice (n = 19). Levels of Igs were log‐transformed before the testing of correlations by Pearson correlation. AU, arbitrary unit

In the previously validated model of CMA used in this study, we confirmed that the induction of allergic symptoms could indeed be mediated by Igs by performing regression analysis using data from nonsensitized and whey‐sensitized mice fed with the control diet (n = 19). This indeed showed a significant positive correlation between the acute allergic skin response and serum levels of IgG1, IgG2a, and IgE (Figure 4A‐C). Similarly, the AUC of the anaphylactic shock score positively correlated with serum levels of IgG1 (*r* = 0.946, *P* < .001), IgG2a (*r* = .893, *P* < .001), and IgE (*r* = 0.603, *P* = .006). To evaluate whether the severity of allergic symptoms in whey‐sensitized mice is correlated with whey‐specific Ig serum levels, regression analysis was performed using data from whey‐sensitized mice (n = 19). The severity of the allergic skin response in whey‐sensitized mice positively correlated with serum levels of both IgG1 (Figure 4D) and IgG2a (Figure 4E), but not with IgE (Figure 4F). Similarly, the AUC of the anaphylactic shock score correlated positively with IgG1 (*r* = 0.509, *P* = .026) and tended to positively correlate with IgG2a (*r* = 0.413, *P* = .079), but not with IgE (*r* = −0.026, *P* = .916).

### 3.4 The AA diet prevents the whey‐induced decrease in FoxP3^+^ cells in the jejunum of whey‐sensitized mice

**Figure 5:**
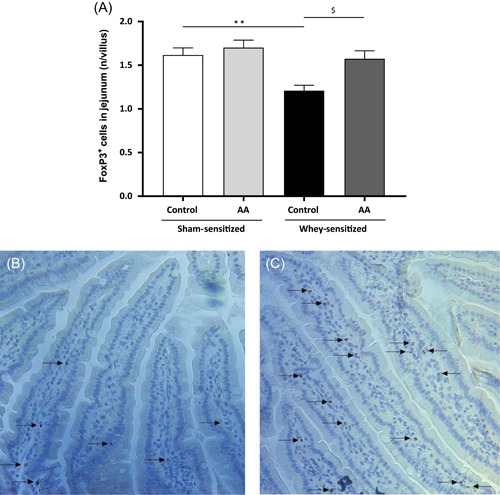
Immunohistochemical staining of Forkhead box P3 (FoxP3) in jejunum sections of mice fed with the control diet and mice fed with the AA diet. A, Cell counts of FoxP3^+^ cells in jejunum sections (n = 9‐10 per group) expressed as number of positive cells per villi. Values are expressed as mean ± SEM. Significant differences between whey‐sensitized and sham‐sensitized mice are indicated by *P < .05 and **P < .01. Differences between whey‐sensitized mice fed with the control diet and those fed with the AA diet are indicated by ^$^ *P* < .05. Differences are analyzed by one‐way ANOVA followed by a Bonferroni post‐hoc test. Representative images of the immunohistochemical staining are shown for (B) whey‐sensitized mice fed with the control diet and (C) whey‐sensitized mice fed with the AA diet. Arrows (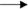) indicate positive intracellular staining for FoxP3. ANOVA, analysis of variance; SEM, standard error of the mean

To determine whether the AA diet affected the development of FoxP3^+^ cells in the small intestines, jejunum sections were stained for FoxP3. Significantly fewer FoxP3^+^ cells were detected in whey‐sensitized mice fed with the control diet compared to diet‐matched sham‐sensitized mice (Figure 5A). Whey‐sensitized mice fed with the AA diet showed significantly more FoxP3^+^ cells than whey‐sensitized mice fed with the control diet (Figure 5A). The AA diet inhibited the whey‐induced decrease in FoxP3^+^ cells, as there was no significant difference between whey‐sensitized mice fed with the AA diet and diet‐matched sham‐sensitized mice (*P* = .468). No significant difference was observed between sham‐sensitized mice fed with the control diet and those fed with the AA diet (Figure 5A). In addition, no differences were observed in villi morphology between mice fed with the AA diet and mice fed with the control diet (Figure 5B,C).

### 3.5 Body weight and food intake is the same for mice fed with the AA diet and those fed with the control diet

**Figure 6:**
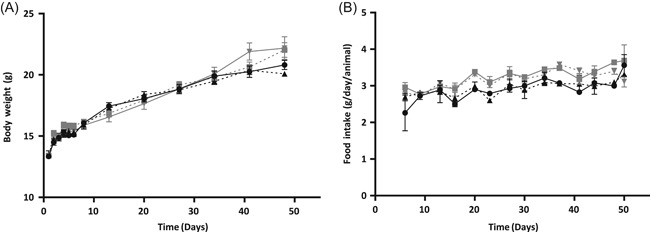
The body weight (A) and food intake (B) of the mice throughout the study protocol. Groups are as follows: 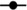 sham‐sensitized mice fed with the control diet (n = 10); 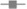 sham‐sensitized mice fed with the AA diet (n = 10); 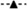 whey‐sensitized mice fed with the control diet (n = 9); 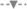 whey‐sensitized mice fed with the AA diet (n = 10). Food intake was measured per cage (n = 2 per group). Differences are analyzed with a one‐way ANOVA followed by a Bonferroni post‐hoc test (A) or a Kruskal–Wallis test with a post‐hoc Dunn's test (B). Values are expressed as mean ± SEM. ANOVA, analysis of variance; SEM, standard error of the mean

To assess whether the compositional differences between the diets interfered with the outcomes by influencing the nutritional status of the mice, body weight and food intake of the mice were monitored throughout the study period. No significant differences were observed in body weight (Figure 6A) or food intake (Figure 6B) between any of the tested groups at any of the time points. Also, total food intake during the study was not different for any of the tested groups. However, there was a strong tendency toward an increase in Δ body weight over the entire study period in whey‐sensitized mice fed with the AA diet compared to those fed with the control diet (*P* = .056), whereas in sham‐sensitized mice, this difference was not observed (*P* = .366; data not shown).

## 4 DISCUSSION

AAFs are used as an option for the dietary management of CMA. It is well‐known that AAFs effectively reduce allergic symptoms and improve growth in children with CMA.^11^, ^19^, ^20^, ^21^, ^22^, ^35^ However, whether AAFs also have the potential to prevent allergic sensitization and/or symptoms associated with CMA is not known. In the present study, a murine model of CMA was used to examine the preventive effects of an AA diet on allergic sensitization and clinical symptoms. This study shows that an AA‐based diet partially inhibits the acute allergic skin response and protects against the whey‐induced anaphylactic symptoms and drops in body temperature. It further indicates that this beneficial effect cannot be explained by a lowered mast‐cell degranulation or decreased production of whey‐specific IgE. However, higher FoxP3^+^ cell counts in the small intestines, combined with lower levels of whey‐specific IgG2a and a tendency toward lower levels of whey‐specific IgG1 in serum may suggest that anti‐inflammatory and tolerizing capacities in AA‐fed mice are enhanced, compared to whey‐sensitized mice fed a protein‐based diet.

Studies in humans reported that an increase in allergen‐specific IgE, IgG1, and IgG2a is typical for IgE‐mediated CMA.^36^ The present study similarly showed that the induction of allergy in mice was associated with an increase in serum levels of allergen‐specific IgE, IgG1, and IgG2a. This indicates that the murine model used in this study is a suitable model for IgE‐mediated CMA, which is described as one of the most commonly occurring allergies among infants.^37^ The preventive effects of the AA diet on allergic symptoms of CMA in mice were accompanied by a strong desirable tendency (*P* = .056) toward more weight gain of AA‐fed whey‐sensitized mice during the study, as compared to whey‐sensitized mice fed with the protein‐based diet. Interestingly, a systematic review by Hill et al^11^ revealed that studies involving infants with IgE‐mediated CMA report comparable clinical improvements following dietary management with an AAF.^38^ This further suggests that the murine model for CMA used in the present study has considerable overlap with the human condition.

The observed beneficial effects of the AA diet on allergic symptoms of allergy were not accompanied by a reduction in serum levels of whey‐specific IgE and mMCP‐1, which are known mediators of allergic symptoms.^39^, ^40^ However, serum levels of IgG2a in whey‐sensitized AA‐fed mice were significantly lower than those levels in whey‐sensitized mice fed with the control diet, and serum levels of IgG1 also tended to be lower in AA‐fed mice. This decrease in levels of IgG2a and IgG1 may, at least partially, be responsible for the observed reduction in allergic symptoms, as multiple studies indicate that allergen‐specific IgG antibodies can have a mediating role in a variety of allergic symptoms.^41^, ^42^, ^43^, ^44^, ^45^, ^46^ For instance, multiple studies showed that both IgG1 and IgG2a are capable of inducing systemic anaphylaxis in mice in Ig‐specific manners, via distinct pathways from that of IgE.^44^, ^47^, ^48^ The present study supports this concept, as serum levels of IgG1 and IgG2a positively correlated with both the acute allergic skin response and the anaphylaxis scores of whey‐sensitized mice. This is in line with earlier observations showing a positive association between serum levels of IgG1 and the acute allergic skin response in CMA mice.^33^

The finding that serum levels of whey‐specific IgE and mMCP‐1 were not affected by the AA diet indicates that the AA diet did not specifically modulate T_H_2‐type immune responses in this model. This is supported by our observation that, in sensitized conditions, both T_H_2‐type IgG1 as well as T_H_1‐type IgG2a were lower, or at least showed a tendency to be lower, in AA‐fed mice. Lower levels of serum IgG in whey‐sensitized mice fed with the AA diet were accompanied by higher numbers of intestinal cells positively stained for FoxP3, which is a marker for regulatory T (Treg) cells.^49^ Several studies have shown that FoxP3^+^ Treg cells can directly and indirectly inhibit the IgG production by B cells,^50^, ^51^, ^52^ which may indicate that the observed differences in IgG1 and IgG2a levels are mediated by FoxP3^+^ Treg cells. In addition, FoxP3^+^ Treg cells are reported to be associated with anaphylactic shock severity in both mice and human, indicating that the increase in FoxP3^+^ Treg cells in AA‐fed whey‐sensitized mice could contribute to the prevention of anaphylactic shock symptoms.^53^, ^54^, ^55^

FoxP3^+^ Treg cells are known to play important roles in modulating immune responses, exerting anti‐inflammatory effects and inducing oral tolerance.^49^, ^56^, ^57^ Hence, the finding that more FoxP3^+^ cells were observed in the jejunum of whey‐sensitized AA‐fed mice than in those mice fed with the control diet may indicate that, in sensitized conditions, AA‐fed mice have enhanced anti‐inflammatory and tolerizing capacities compared to mice fed the control diet. Lower levels of both T_H_2‐ and T_H_1‐type IgGs as found in whey‐sensitized AA‐fed mice also indicate that the AA diet has an anti‐inflammatory effect. To support this indication, future studies on the preventive effects of AAFs on CMA should examine systemic and intestinal levels of proinflammatory and anti‐inflammatory cytokines, as well as T_H_1 and T_H_2 cytokines after allergen challenge. In addition, levels of anti‐inflammatory chemokine galectin‐9 should be measured in future studies. Galectin‐9 is shown to reduce allergic symptoms by sequestering IgE and by inducing T_reg_ cell polarization.^58^, ^59^, ^60^ Thus, it can be hypothesized that the observed increase in T_reg_ cells in AA‐fed whey‐sensitized mice, as well as the AA diet‐driven reduction of allergic symptoms in absence of reduced IgE levels is mediated by galectin‐9. The capacity of an AAF to reduce proinflammatory cytokine and chemokine production in inflammatory conditions has been demonstrated previously. For instance, it is shown that intake of an AAF with an AA composition highly similar to that of the AA diet used in this study (ie, Neocate) reduces colonic inflammatory status in infants with non‐IgE‐mediated CMA.^26^ This was evidenced by lower baseline levels of proinflammatory cytokines TNF‐α and IL‐6 in biopsy supernatants of the allergic infants with the AAF in their diet compared to those without the AAF in their diet. Furthermore, ex vivo treatment of these biopsies with the AAF reduced the production of T_H_2 cytokines (a.o. IL‐4 and IL‐13) and various proinflammatory cytokines, indicating that the AAF exerts direct anti‐inflammatory effects.^26^ This indication is supported by a study showing that treatment of LPS‐stimulated human peripheral blood mononuclear cells with the AA portion of the previously mentioned AAF reduces TNF‐α production as well as C‐X‐C motif chemokine ligand 8‐induced neutrophil chemotaxis.^27^ Interestingly, the latter study also demonstrated that the free AAs in the AAF are, at least partially, responsible for the observed anti‐inflammatory effects. As free AAs are known to have AA‐specific and diverse functions, including immunomodulatory functions, it can be speculated that the specific AA composition of the diet used in this study may contribute to the observed effects.

The AA diet used in this study contained a relatively high amount of free glutamine and glycine, which are both considered conditionally essential AAs. In terms of millimole AA per kg of feed, glutamine and glycine were the two most abundant AAs in the AA diet, together comprising more than 20% of all AAs present. These two free AAs are known to have a range of immunomodulatory effects. For instance, free glutamine supplementation is shown to decrease the production of proinflammatory cytokines while increasing anti‐inflammatory cytokines, to improve epithelial barrier function and to prevent inflammation‐induced intestinal damage in a variety of in vitro, in vivo, and ex vivo immune models.^61^, ^62^, ^63^, ^64^, ^65^ In a mouse model of colitis, dietary supplementation of glutamine decreased levels of IgG in lavage fluid and fully prevented the colitis‐induced decrease in blood FoxP3^+^ cells and decrease in FOXP3 messenger RNA in mesenteric lymph nodes, similar to the effects of the AA diet observed in this study.^66^ Similar to glutamine, free glycine supplementation is also shown to reduce levels of proinflammatory cytokines and to increase levels of regulatory cytokines in several in vitro and in vivo immune models.^67^, ^68^, ^69^ In addition, oral exposure to free glycine prevented the onset of CMA in mice, which was accompanied by a reduction in serum levels of IgG1 and IgG2a.^70^ Based on these findings, it can be speculated that the immunomodulatory effects of glutamine and glycine contribute to the observed preventive effects of the AA diet on symptoms of CMA.

The present study did not find differences in any of the tested clinical, mechanistic, and nutritional parameters between sham‐sensitized mice fed with the AA diet and sham‐sensitized mice fed with the control diet. Although the absence of differences remains to be confirmed at the level of cytokine production, these findings may suggest that the intake of an AA‐based diet does not lead to immune disturbances in healthy individuals. This is in accordance with clinical studies reporting that intake of AAFs is safe, well‐tolerated and without any indications for long‐term effects.^22^, ^23^, ^24^

In the murine model used in the present study, a soy‐protein‐based diet was used as a control diet, as the diets should be free of cow's milk protein for the investigation of CMA. A variety of studies indicate that a soy protein‐based diet exhibits anti‐inflammatory effects.^71^, ^72^, ^73^ For instance, soy food intake was inversely correlated with levels of several proinflammatory cytokines, including IL‐6, in blood of Chinese women.^71^ As proinflammatory cytokines drive allergic sensitization, the soy‐based control diet used in this study may also exert preventive effects on allergic sensitization. Thus, as the present study evaluated the preventive effects of an AA‐based diet through comparison with a soy protein‐based diet, the observed effects of the AA‐based diet on the prevention of CMA may be underestimated.

In summary, the present study showed that intake of an AA‐based diet before and during sensitization prevented, at least partially, the development of allergic symptoms in allergen‐challenged whey‐sensitized mice. The exact mechanism underlying the observed effects has yet to be revealed, however, this study suggests the involvement of FoxP3^+^ Treg cells and IgG2a antibodies, and potentially also IgG1 antibodies. Although confirmation in humans is critical as findings in mice do not always directly translate to humans, the observed beneficial effects in whey‐sensitized mice, combined with the absence of effects on growth and development in sham‐sensitized mice indicates that AAFs for the prevention of food allergies may be an interesting concept that warrants further research.

## CONFLICT OF INTERESTS

SW, LH, RL, and AHa are employed at Danone Nutricia Research. JG is partly employed at Danone Nutricia Research. All other authors report no potential conflict of interests.

## Data Availability

The datasets generated during and/or analyzed during the current study are available from the corresponding author on reasonable request.
